# Database: web application for visualization of the cumulated RNAseq data against the salicylic acid (SA) and methyl jasmonate (MeJA) treatment of *Arabidopsis thaliana*

**DOI:** 10.1186/s12870-020-02659-y

**Published:** 2020-10-02

**Authors:** Dong U Woo, Ho Hwi Jeon, Halim Park, Jin Hwa Park, Yejin Lee, Yang Jae Kang

**Affiliations:** 1 Division of Bio & Medical Big data department (BK4 Program) at Gyeongsang National University, Jinju, Republic of Korea; 2 Division of Life Science Department at Gyeongsang National University, Jinju, Republic of Korea

**Keywords:** Salicylic acid, Jasmonic acid, Web-application, Arabidopsis, RNAseq, Database

## Abstract

**Background:**

Plants have adapted to survive under adverse conditions or exploit favorable conditions in response to their environment as sessile creatures. In a way of plant adaptation, plant hormones have been evolved to efficiently use limited resources. Plant hormones including auxin, jasmonic acid, salicylic acid, and ethylene have been studied to reveal their role in plant adaptation against their environment by phenotypic observation with experimental design such as mutation on hormone receptors and treatment / non-treatment of plant hormones along with other environmental conditions.

With the development of Next Generation Sequencing (NGS) technology, it became possible to score the total gene expression of the sampled plants and estimate the degree of effect of plant hormones in gene expression. This allowed us to infer the signaling pathway through plant hormones, which greatly stimulated the study of functional genomics using mutants. Due to the continued development of NGS technology and analytical techniques, many plant hormone-related studies have produced and accumulated NGS-based data, especially RNAseq data have been stored in the sequence read archive represented by NCBI, EBI, and DDBJ.

**Description:**

Here, hormone treatment RNAseq data of Arabidopsis (Col0), wild-type genotype, were collected with mock, SA, and MeJA treatments. The genes affected by hormones were identified through a machine learning approach. The degree of expression of the affected gene was quantified, visualized in boxplot using d3 (data-driven-document), and the database was built by Django.

**Conclusion:**

Using this database, we created a web application (http://pgl.gnu.ac.kr/hormoneDB/) that lists hormone-related or hormone-affected genes and visualizes the boxplot of the gene expression of selected genes. This web application eventually aids the functional genomics researchers who want to gather the cases of the gene responses by the hormones.

## Background

Plants are sessile organisms that adapt to numerous external stimuli in order to change them into favorable conditions or survive in adverse conditions of their surrounded environment. For this purpose, plants produce small amounts of endogenous regulators, such as phytohormones, in the cells of leaves, stems, or roots, and transport them to other parts to use them to control plant metabolism [1]. Hence, the even small amount of plant hormone is so important for plant metabolism and many scientists have studied the function, synthesis, transport, and signaling pathways of plant hormones through the genetic approaches in a model plant, *A. thaliana*, using various measurements. As a result, some roles of the plant hormones have been well studied. For example, auxins, gibberellins, and cytokinins are mainly involved in plant growth, ethylene in fruit ripening, and abscisic acid in seed dormancy [2], jasmonic acid (JA) induces pest resistance [3], salicylic acid (SA) induces pathogen resistance and plant systemic resistance [4], and brassinosteroids in vascular bundle differentiation [5] and strigolactone leads the soil microbial response [6].

As such, the genetic approaches of *A. thaliana* using mutants could reveal the function of genes through the changes in phenotype that occur after gene loss/gain estimating the roles of plant hormones and their signaling pathways. Besides the genetic approaches that are on the limited number of genes, the microarrays were used to analyze transcriptional expressions of large gene set to understand more complex gene expression pathways. Moreover, with the continuous development of next-generation sequencing (NGS) technology and analytic methodology, RNA-seq truly revolutionized the detection of transcriptional expression of whole genes and other regulatory elements such as small RNAs [7]. Based on these technological advances, gene expression analysis of various growth stage and hormone concentrations of cytokinin [8], and auxin and abscisic acid [9] were carried out with regard to the phenotypes of germination, leaf formation, rosette growth, flowering, and etc.. As the transcriptional profiles of the tissues on the various conditions are so dynamic that many research groups are continuously producing RNAseq data to determine how plant hormones affect plants and which genes are associated with them.

Meanwhile, the researchers continuously revisit or request the published raw data for other analytic purposes or regeneration of the published results using newly developed methods. It became so common for the researchers to deposit their research materials; the raw NGS data, the analyzed data, and the metadata of experimental information to the public databases; NCBI, EBI, and DDBJ [10, 11]. Currently, a large amount of NGS data is disclosed through public databases without special permission. As the uploaded NGS data exploded, the researchers tried to derive new meanings using the original biological knowledge and the deposited NGS data; however, the metadata with the detailed information of samples are frequently inconsistent or absent, so it became highly laborious to correct the metadata and to list up the comparable data with regard to data generation methods. Moreover, it was difficult to handle the large amounts of deposited NGS data without trained bioinformaticians.

Therefore, we collected the RNAseq data of plant hormone treatment researches from NCBI sequence read archive (SRA) and manually examined the metadata information. Well-examined data were processed into the expression values in the normalized unit such as trimmed mean of M-values normalization (TMM), visualized in grouped boxplots and description panels on the web application. We targeted the SA, JA and their mock treatment for our database construction as they are currently well-deposited to form large datasets compared to other hormones. The expression levels of genes are shown in a grouped boxplot using the d3 visualization platform [12]. Moreover, we applied a machine learning algorithm, random forest, to classify the samples into JA, SA, and mock treatments. The resulting model clearly indicates important features (genes) for the classification that would be well associated with the treatments and this result is also listed in our web application.

Our application would be a useful application for the researchers who are interested in the hormone responses of genes in *A. thaliana* genome, especially for the SA and JA. Moreover, the selected gene set that is distinctly up- and down-regulated would be strong candidate genes participating in SA and JA signaling pathways. Furthermore, exponentially increasing RNAseq data on other hormone treatments would be updated yearly on our database.

### Construction and content

#### Workflow for the DB construction

**Fig. 1:**
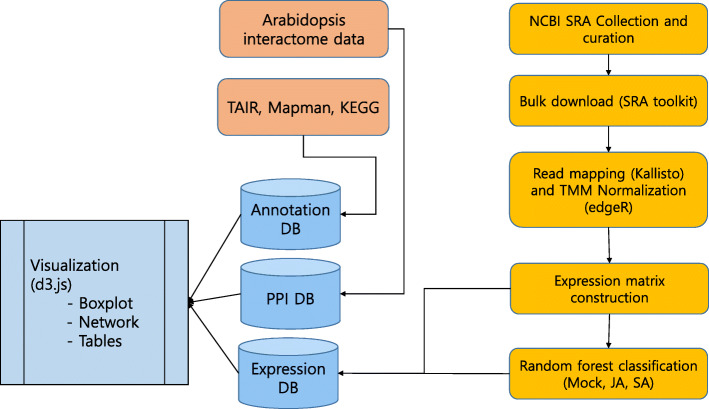
Flowchart for the data preparation and development of Arabidopsis hormone DB

For the construction of our SA/JA-induced gene expression DB, we built a simple scheme for data preparation, DB construction, and web-application design (Fig. 1). The data preparation step consists of the examination of NCBI-deposited RNAseq studies to determine the target dataset to host. For DB construction step, we implemented the sqlite3 import of well-prepared data and public data of Mapman, KEGG, Protein-protein interaction of *A. thaliana*. Finally, we designed the user-interface of our web-application that would facilitate knowledge mining.

#### Sample collection from NCBI SRA

We tried to collect metadata information of RNAseq data that are deposited in NCBI SRA. The metadata should possess descriptions of the samples such as tissue, treatment, ecotype, induced mutation, sequencing platform, and so on. However, not every study contains enough information that actually allows the comparison among samples. For this reason, we manually examined the metadata and filter out the SRA entries which don’t possess essential information. We defined the essential information to fill the following columns; Study ID, Run ID, Assay type, Genotype, Treatment, Developmental stage, Tissue, and Layout (Supplementary Table 1). A total of 1000 hormone-related RNAseq studies were collected (Supplementary Figure 1A) and the experiments of SA and JA treatments were dominated. We limited the “Genotype” to Col0, and the “Treatment” to control (245), SA_1.0 mM(224), SA_2mM(1), MeJA_0.1 mM (224), JA_1mM (1) where the sampling times after treatment were from 15 min to 72 h (Supplementary Figure 1B). We excluded multi-treatment experiments. For the control, we collected the experiments treated with water or DMSO. We also narrowed the studies to the leaf tissue. The developmental stages were restricted to the samples that are labeled as ‘5-w-old plant’, ‘3w-old’, ‘Vegetative growth’. After this examination, we could list up 695 experiments with SRR ID which consisted of three SRA studies; SRP031882, SRP112501, and SRP125543 [13, 14].

### Construction of expression matrix construction, analysis, and storage

**Fig. 2:**
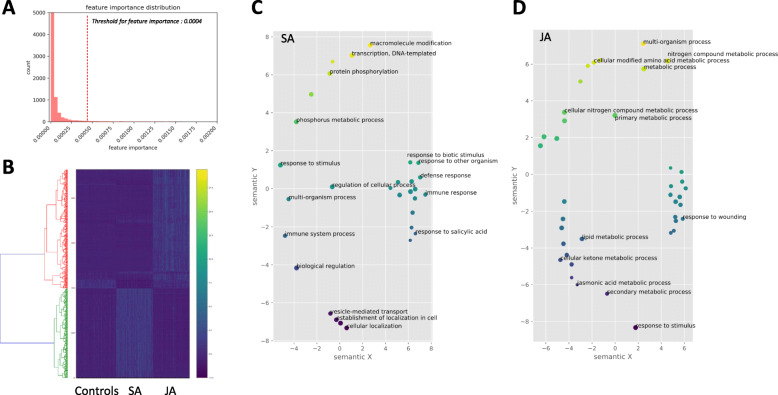
Random forest based candidate gene extraction. **a** Distribution of the feature importance values for the classification among control, SA, and JA. **b** Gene expression heatmap of the selected candidate genes. **c** GO annotations for SA specific transcripts and **d** JA specific transcripts

The collected SRA studies were downloaded using SRA-toolkit [15] based on the collected Run ID. After converting the SRA files into the text-based NGS reads format, Fastq, we mapped the reads onto the reference coding sequences of *A. thaliana* (Araport 11) [16] and calculated the expression values (trimmed mean of M-values normalization, TMM) of transcripts using EdgeR and software Kallisto [17, 18]. We applied the random forest (RF) classification scheme to classify the samples based on the treatments; Control, JA, SA using a scikit-learn software package [19]. The RF training applied 100-time iteration, to retrieve the distribution of feature importances for each transcript. From the RF training, each gene will be assigned 100 feature importances that explain how important the genes for the classification of control, SA, and JA groups. We averaged the 100 feature importances and plotted the histogram of them (Fig. 2a). We selected the threshold of feature importance value as 0.0004. The threshold was determined to have the top 1% of the important genes from the distribution. A total of 463 candidate genes were extracted from RF model training. The filtered transcripts showed distinct expression patterns according to the treatments (Fig. 2b). The GO annotation using REVIGO [20] shows SA and JA specific terms; “response to salicylic acid” and “jasmonic acid metabolic process”, respectively. SA specific gene set additionally contains “immune system response” and “leaf senescence”. JA specific gene set includes “response to wounding” and “hormone metabolic process” (Fig. 2c and d). The total expression values for genes and the list of candidate genes from RF training were stored in the sqlite3 database.

#### Web-application construction and visualization

**Fig. 3:**
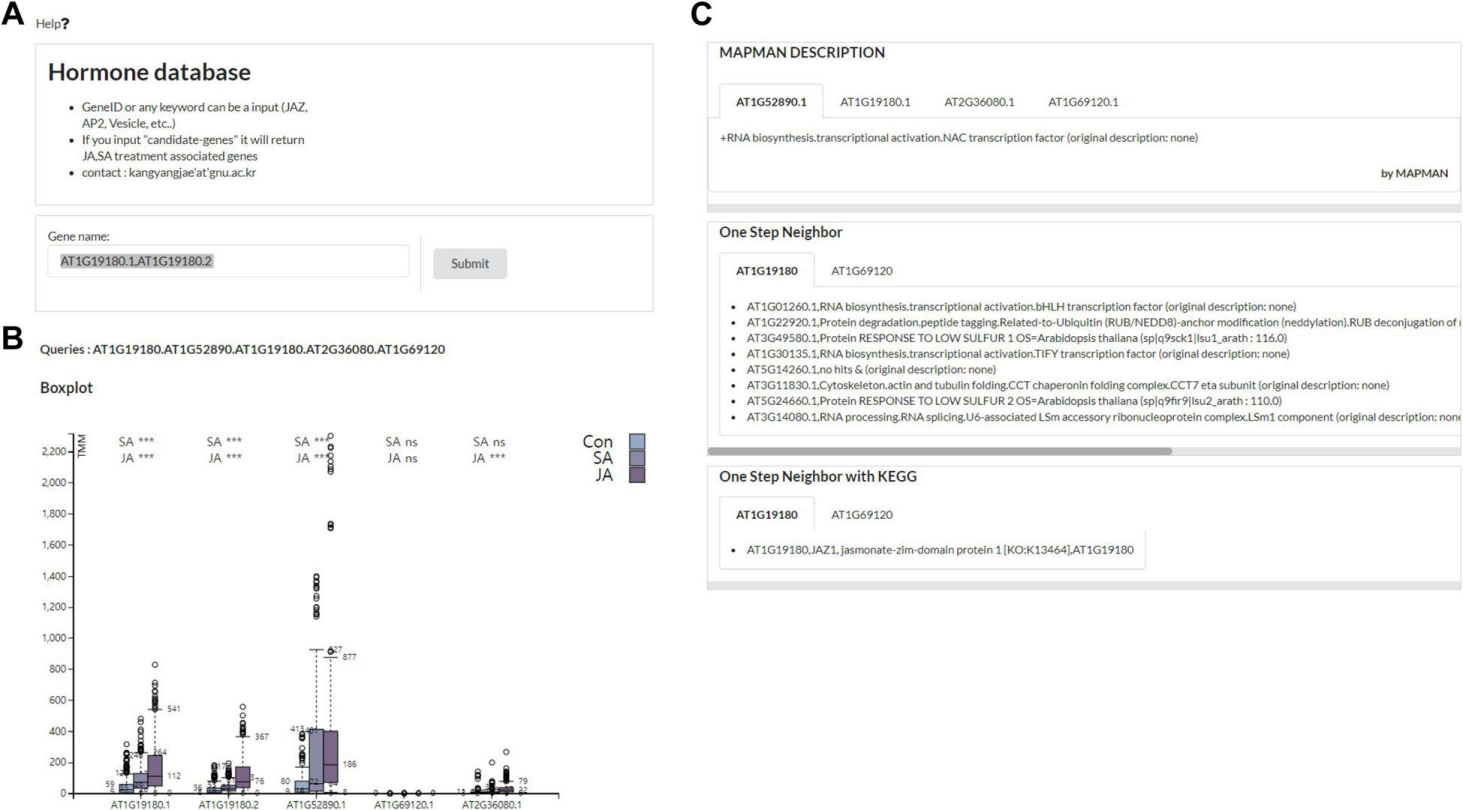
User-interface of our web-application; **a** Gene query input panel. **b** Boxplot showing gene expression distribution of control, SA, and JA treatment. **c** Gene descriptions of Mapman and the one-step neighbor based on PPI network and KEGG

**Fig. 4:**
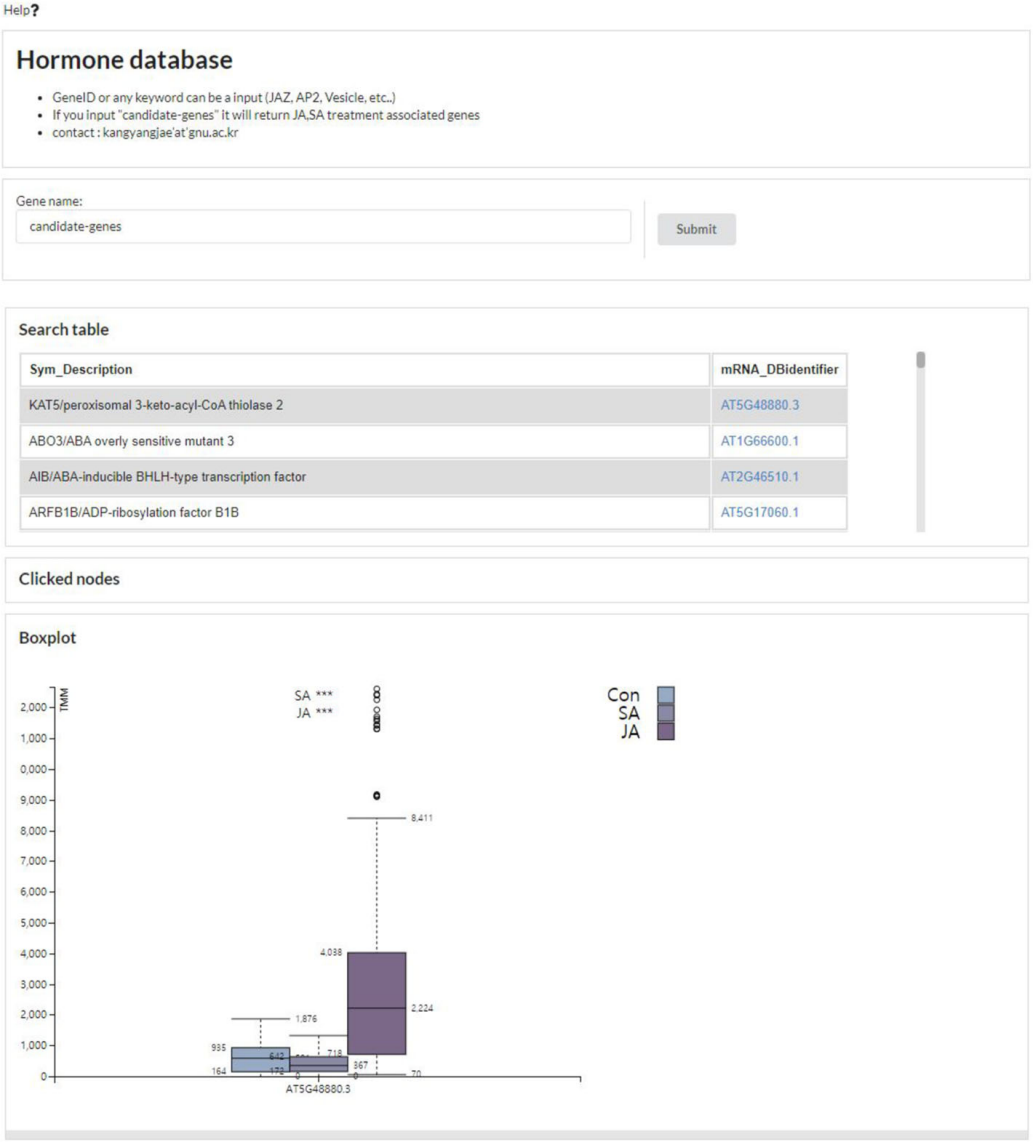
List up the candidate genes from the random forest analysis for exploring the interesting genes

Based on the Django web framework (https://www.djangoproject.com) and d3.js (https://d3js.org/) [12], we could visualize the results of gene expressions and candidate genes on the web-based application. Moreover, the design of the web-pages is built using semantic UI (https://semantic-ui.com/). For the query form, the users can input transcript ID (eg. AT1G19180.1) and also input gene ID (eg. AT1G19180) Moreover, users can input any searching keywords such as “JAZ”, “Vesicle”, “Transcription” and so on (Fig. 3a). We tried to display the query associated information; such as transcript expression in grouped boxplot (Fig. 3b), gene description [16], KEGG pathway [21], Mapman ontology [22], Arabidopsis interactome [23] (Fig. 3c). In addition, in the case of boxplot, each t-test significance between control and treatment is displayed at the top of the grouped boxplots. We used Mapman ontology to intuitively provide the gene classification information of the transcripts. Moreover, we added the neighbor information of PPIN and KEGG pathways to present rich information of the query gene. To list up the result of candidate gene extraction from RF training, we allowed searching keyword “candidate-genes”. If user input “candidate-genes”, it will list up the distinctly expressed genes in the Search table panel and can be clicked to see the expression pattern and the many annotations directly (Fig. 4).

## Utility and discussion

Since the development of NGS technology, many genomic studies based on this technology have been conducted in the model plant, *A. thaliana*. In NCBI SRA, a total of 74 terabytes of NGS data is accumulated for *A. thaliana*. We examined and collected the NGS data which have been studied on the plant hormones and explored treatment-dependent candidate genes through RF training-based feature selection scheme. To make them into web-application, we utilized a web framework Django that allows us to manage databases, and to host our webpage which provides useful functionalities; the visualization of their gene expression and network, the classification, and description of genes. As the RNAseq deposit in NCBI SRA for other hormone treatments are also increasing we are planning the annual updates and renewal of our web-application to expand the usability. We expect that many researchers can find the expression patterns of their target genes with regards to JA and SA treatments and retrieve new interesting genes from our candidate gene list without special knowledge of bioinformatics.

## Conclusions

In this work, we have developed a searchable database focused on RNAseq data of *A. thaliana* subjected to SA and JA pathways, leading to a web application improved with machine learning through large datasets. The database will be updated annually to account the cumulative RNAseq data in NCBI. Overall this new web platform will help plant researchers find easily hormone responding genes in transcriptional profiles.

## Supplementary information

**Additional file 1.**

**Additional file 2.**

## Data Availability

The application’s web address is http://pgl.gnu.ac.kr/hormoneDB/. The datasets analysed during the current study are listed in Supplementary Table 1.

## Abbreviations

GO: Gene ontology
JA: Jasmonic acid
KEGG: Kyoto encyclopedia of genes and genomes
NCBI: National Center for Biotechnology Information
NGS: Next generation sequencing
RF: Random forest
SA: Salicylic acid
SRA: Sequence read archive
TPM: Transcripts per million
