# Supplementary figures and images for: Database: web application for visualization of the cumulated RNAseq data against the salicylic acid (SA) and methyl jasmonate (MeJA) treatment of Arabidopsis thaliana
Source: BMC Plant Biol. 2020 Oct 2;20:453. doi: 10.1186/s12870-020-02659-y (PMC7532101; doi:10.1186/s12870-020-02659-y)

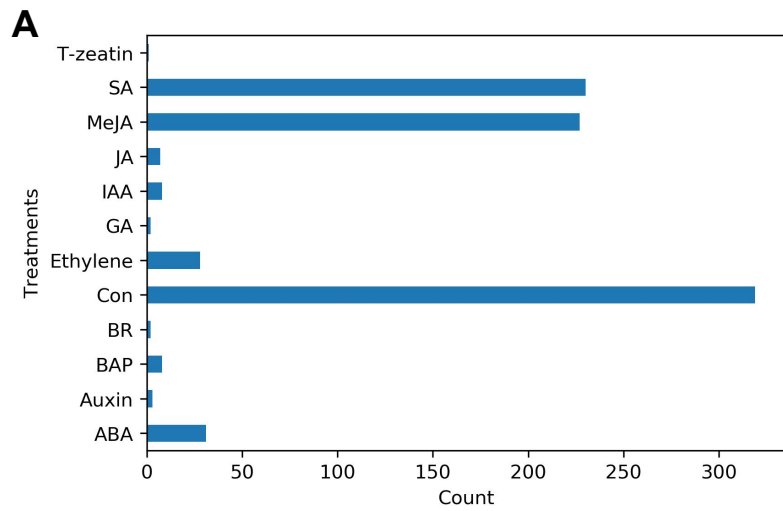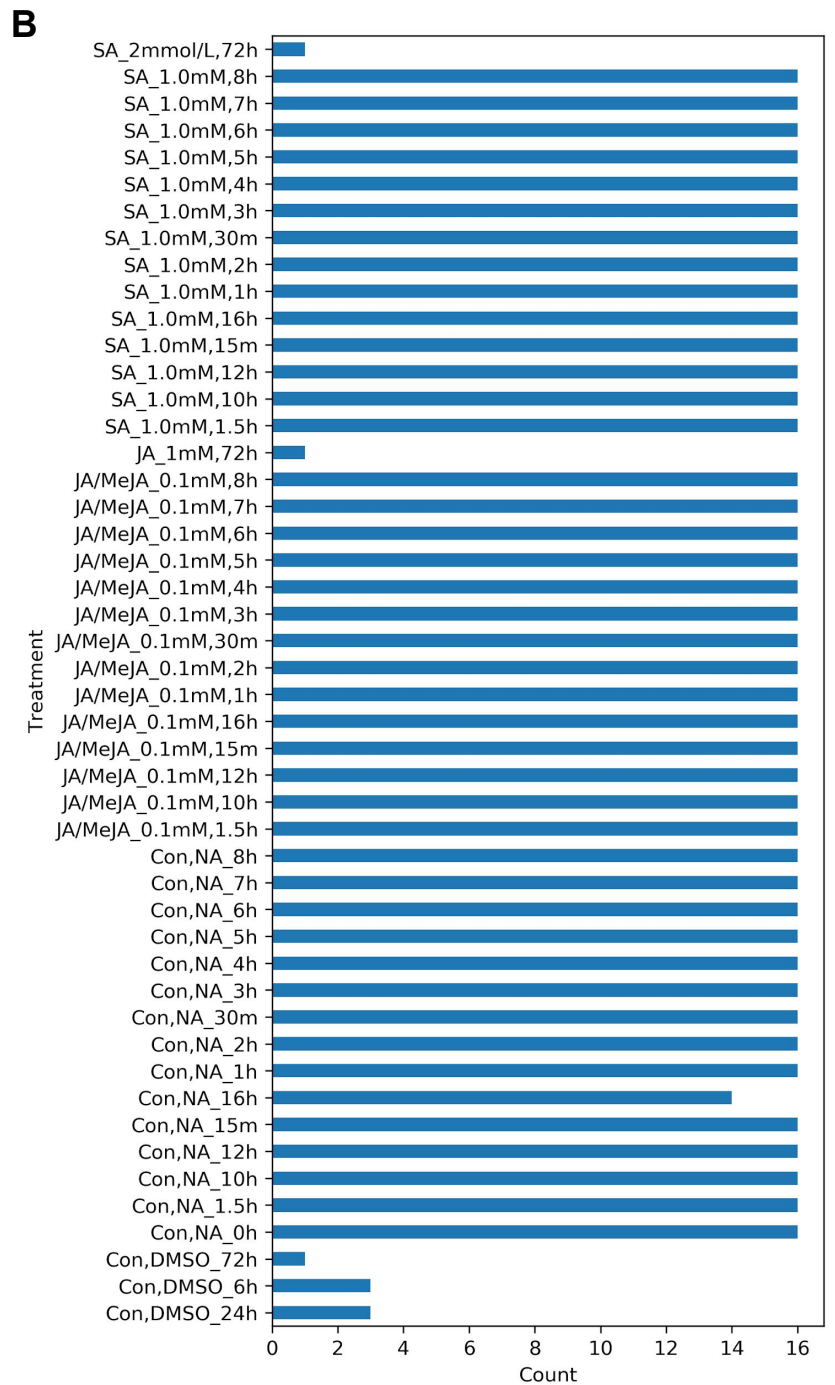

Supplement: Supplementary file 1 — Additional file 1. [file 12870_2020_2659_MOESM1_ESM.pdf]
